# Boron‐Doped Zinc Oxide Electron‐Selective Contacts for Crystalline Silicon Solar Cells with Efficiency over 22.0%

**DOI:** 10.1002/smsc.202400168

**Published:** 2024-09-22

**Authors:** Zheng Li, Anzhi Xie, Qingxian Nong, Yiwei Sun, Haihuai Cai, Zhexi Chen, Jian He, Pingqi Gao

**Affiliations:** ^1^ School of Materials Shenzhen Campus of Sun Yat‐sen University No. 66, Gongchang Road Shenzhen Guangdong 518107 P. R. China; ^2^ Institute for Solar Energy Systems Guangdong Engineering Technology Research Center for Sustainable Photovoltaic Technology and Equipment State Key Laboratory of Optoelectronic Materials and Technologies Sun Yat‐sen University Guangzhou 510275 P. R. China

**Keywords:** boron‐doped zinc oxides, crystalline silicon solar cells, electron‐selective contacts, passivations

## Abstract

The exploration of wide‐bandgap metal compound films with excellent passivation and contact properties on crystalline silicon (c‐Si) surface, as alternatives to traditional‐doped Si thin films, holds significant promise for the future enhancement of c‐Si solar cell efficiency. Herein, conductive boron‐doped zinc oxide (ZnO:B) films are deposited by atomic layer deposition (ALD) process and investigated as electron‐selective contacts, in combination with a thin SiO_*x*_ passivating interlayer. This combination demonstrates a relative low contact resistivity of ≈2 mΩ cm^2^ and improved passivation quality. Further application of this SiO_*x*_/ZnO:B stack as a full‐area electron‐selective passivating contact in proof‐of‐concept n‐type c‐Si solar cells results in a satisfactory power conversion efficiency of over 22.0%. This electron‐selective passivating contact structure, prepared via low‐temperature, simplified, and the compositionally controlled ALD process, offers a promising pathway for the development of high‐efficiency and low‐cost c‐Si solar cells.

## Graphical abstract

Herein, conductive boron‐doped zinc oxide (ZnO:B) films deposited by atomic layer deposition are investigated as electron‐selective contacts together with ultrathin SiO_*x*_ passivating interlayer, demonstrating a relative good passivation and contact properties. A satisfied power conversion efficiency of over 22.0% is demonstrated while using this SiO_*x*_/ZnO:B electron‐selective contact in proof‐of‐concept c‐Si solar cells.

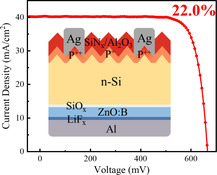

## 1 Introduction

Photovoltaic (PV) technology is considered one of the crucial methods for achieving environmental sustainability. As the dominant PV technology, the primary development goals for crystalline silicon (c‐Si) solar cells are to enhance the power conversion efficiency (PCE) and also reduce costs.^[^ ^1^ ^]^ One recognized effective approach is the development of passivating contact technology, which incorporates a thin passivation layer and a thin carrier selective transport layer between c‐Si and metal electrodes to achieve effective carrier separation and collection at c‐Si surface.^[^ ^2^ ^]^ For instance, n‐type tunneling oxide passivation contact (TOPCon) solar cells can achieve production PCEs exceeding 25% using a SiO_*x*_ layer as passivation layer and a phosphorus‐doped poly‐Si as electron‐selective transport layer on the rear side.^[^ ^3^ ^]^ Si heterojunction (SHJ) solar cells have demonstrated laboratory PCE of over 26.8% using intrinsic a‐Si:H layer as the passivation layer and boron‐/phosphorus‐doped a‐Si:H layer as the hole‐/electron‐selective transport layer on both sides.^[^ ^4^ ^]^ Despite the high PCEs achieved by both TOPCon and SHJ solar cells, their exceptional carrier‐selective transport performance relies heavily on doped Si films. SHJ solar cells also require a high‐quality intrinsic a‐Si:H layer to obtain excellent interface passivation properties. The use of these Si films leads to unnecessary optical parasitic absorption loss, which limits the short‐circuit current density (*J*_SC_) of the solar cells. Additionally, these films are highly dependent on specialized equipment and involve toxic flammable gases such as phosphine and silane.^[^ ^5^, ^6^ ^]^ Therefore, to further improve the PCE of c‐Si solar cells and reduce the manufacturing costs, it is essential to develop alternative carrier‐selective transport materials continuously.

Recently, the utilization of functional compound materials, such as oxides, nitrides, fluorides, to replace traditional highly doped Si film in c‐Si solar cells and form dopant‐free carrier‐selective passivating contacts with c‐Si has garnered significant attention.^[^ ^7^ ^]^ In these solar cells, the selective transport of carriers is primarily achieved through band alignment with c‐Si. For instance, an upward band bending on c‐Si surface is typically expected to transport holes when in contact with high‐work function transition metal oxides (TMOs).^[^ ^8^ ^]^ Conversely, a downward band bending or small conduction band offset with c‐Si is anticipated to transport electrons using low‐work function compound materials.^[^ ^9^ ^]^ Currently, the PCEs of c‐Si solar cells based on TMO hole‐selective contacts (HSCs) reached 23.8%, while those with oxides’ electron‐selective contacts (ESCs) reached to 23.1%.^[^ ^10^ ^]^

Unlike HSCs, which are mainly limited to TMOs, for example, MoO_*x*_,[^10^, ^11^] V_2_O_*x*_,^[^ ^12^ ^]^ WO_*x*_,^[^ ^13^ ^]^ and MoN_*x*_,^[^ ^14^ ^]^ ESCs offer a wider range of material options. For instance, metal fluorides with low work function, such as LiF_*x*_,^[^ ^15^ ^]^ MgF_*x*_,^[^ ^16^ ^]^ AlF_*x*_,^[^ ^17^ ^]^ EuF_*x*_,^[^ ^18^ ^]^ and SrF_*x*_^[^ ^19^ ^]^ have been successfully utilized as ESCs for n‐type c‐Si solar cells, achieving PCEs of over 20%. Additionally, metal oxides and nitrides with negligible conduction band offsets with c‐Si, including MgO_*x*_,^[^ ^20^ ^]^ TiO_*x*_,[^10^, ^21^] TiN_*x*_,^[^ ^22^ ^]^ TaN_*x*_,^[^ ^23^ ^]^ TiO_*x*_N_y_,^[^ ^24^ ^]^ SrO_*x*_,^[^ ^25^ ^]^ and Li_3_N,^[^ ^26^ ^]^ have been explored as ESCs. Despite the various materials reported for ESCs, their final PV output performance remains unsatisfied and is still lower than that of SHJ and TOPCon solar cells. Among these ESC materials, ZnO stands out due to its excellent photoelectric properties, for example, wide bandgap, high carrier mobility, and suitable energy level.^[^ ^27^ ^]^ Zhong et al. realized a PCE of 21.4% by depositing a thick ZnO_*x*_ film on the rear side to mitigate plasmonic absorption losses.^[^ ^28^ ^]^ Xie et al. fabricated a bifacial c‐Si heterojunction solar cell with an efficiency of 21.2% under front‐side irradiation and 20.4% under rear‐side irradiation using atomic layer deposition (ALD)‐ZnO_*x*_ as a transparent ESCs on the front side.^[^ ^29^ ^]^ Intrinsic ZnO film can exhibit n‐type semiconductor characteristics due to the presence of oxygen vacancies and interstitial Zn ions. This electrical property can be further enhanced by donor doping, such as B, Al, Ga, H doping.^[^ ^30^ ^]^ For example, a high carrier mobility of 38.7 cm^2^ V^−1^ s^−1^ can be achieved by direct current magnetron sputtering using Al as dopant (ZnO:Al).^[^ ^31^ ^]^ ALD‐deposited B‐doped ZnO (ZnO:B) film with a doping fraction of 0.016 shows a low resistivity of 3.5 mΩ cm.^[^ ^32^ ^]^ Regarding the application of doped ZnO film in c‐Si solar cells, Ding et al. reported a solution‐processed ZnO:Al film as ESC, achieving a PCE of 18.46% when combined with an intrinsic a‐Si:H layer as passivating interlayer.^[^ ^33^ ^]^ This PCE is further improved to 19.5% by Zhong et al. using SiO_*x*_/ZnO:Al as ESCs and intrinsic a‐Si:H as passivating interlayer.^[27d]^

In this study, we have demonstrated the feasibility of using ALD‐deposited ZnO:B films as ESCs in n‐type c‐Si solar cells. By properly controlling the B doping concentration, the ZnO:B film can exhibit excellent electrical properties with resistivity as low as 3.0 mΩ cm. Combined with a thin SiO_*x*_ passivating interlayer, the passivation and contact performance of the n‐Si/SiO_*x*_/ZnO:B contact were investigated and optimized. The underlying mechanism was elucidated through detailed composition and interface characterization. When applying this n‐Si/SiO_*x*_/ZnO:B stack as ESCs in proof‐of‐concept n‐type c‐Si solar cells, we achieved PCEs of over 22.0%. This approach demonstrates an effective way to design high‐performance and stable ESCs using a simplified fabrication process.

## 2 Results and Discussion

### 2.1 Characterization of ZnO:B Film

**Figure 1:**
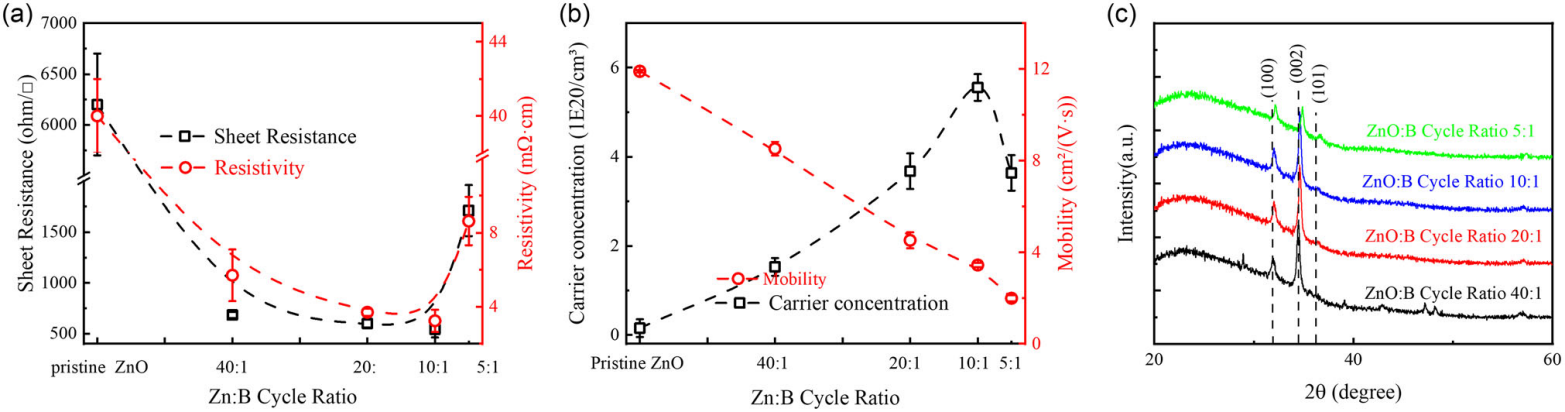
Electrical and structural properties of ZnO:B film. a) Sheet resistance, resistivity, b) carrier concentration and mobility of ZnO:B films with different doping ratios, and c) XRD spectrum of ZnO:B films with different doping ratios.

ZnO:B films were deposited using the ALD method in supercycle mode. The growth rate at different B doping cycle ratios is summarized in Figure S1, Supporting Information, showing a reduced deposition rate as the doping ratio increases. This reduction is due to the inhibition of ZnO growth after a triisopropyl borate (TIB) pulse, which persists for several cycles following the TIB pulse.^[^ ^34^ ^]^ The electrical properties of ZnO:B films at different B doping cycle ratios are summarized in **Figure** **1**. The conductivity of the ZnO:B film improves significantly when the boron doping ratio is increased to 10:1, achieving a best resistivity of about 3.0 mΩ cm (compared with pristine ZnO film with resistivity of about 40.0 mΩ cm). Further increasing the doping ratio degrades the conductivity, potentially attributed to the dopant clustering, formation of oxides, or metastable phases, which render the dopant inactive.^[^ ^35^ ^]^ This trend is consistent with the variation in carrier concentration in ZnO:B films (Figure 1b). At low doping concentrations, the introduction of B atoms instead of Zn atoms effectively provides additional free carriers, thereby increasing the carrier concentration. However, excessive doping leads to B atoms filling interstitial sites and forming B–O complexes, which reduce the concentration of oxygen vacancies in the film, as evidenced by the X‐ray photoelectron spectroscopy (XPS) results in Figure S2, Supporting Information.^[^ ^35^ ^]^ The electron mobility of the ZnO:B film, characterized in Figure 1b, also shows that increased B doping affects the crystal quality of the ZnO film. This results in lattice deformation and the introduction of defects, grain boundaries, etc. The X‐ray diffraction (XRD) characterization in Figure 1c, showing that as the doping concentration increases, the position of the peak shifts, leads to a decrease in mobility.^[^ ^34^ ^]^ Additionally, the transmittance curve of ZnO:B at different doping ratios is shown in Figure S3, Supporting Information, showing that as B doping ratio increases, the transparency of ZnO:B film increases significantly. The blueshift in the absorption edge of the B‐doping ZnO at the short wavelength (<500 nm) should attributed to the Burstein–Moss effect and the bound exciton effect, which results from exciton–impurity interactions.^[^ ^36^ ^]^

**Figure 2:**
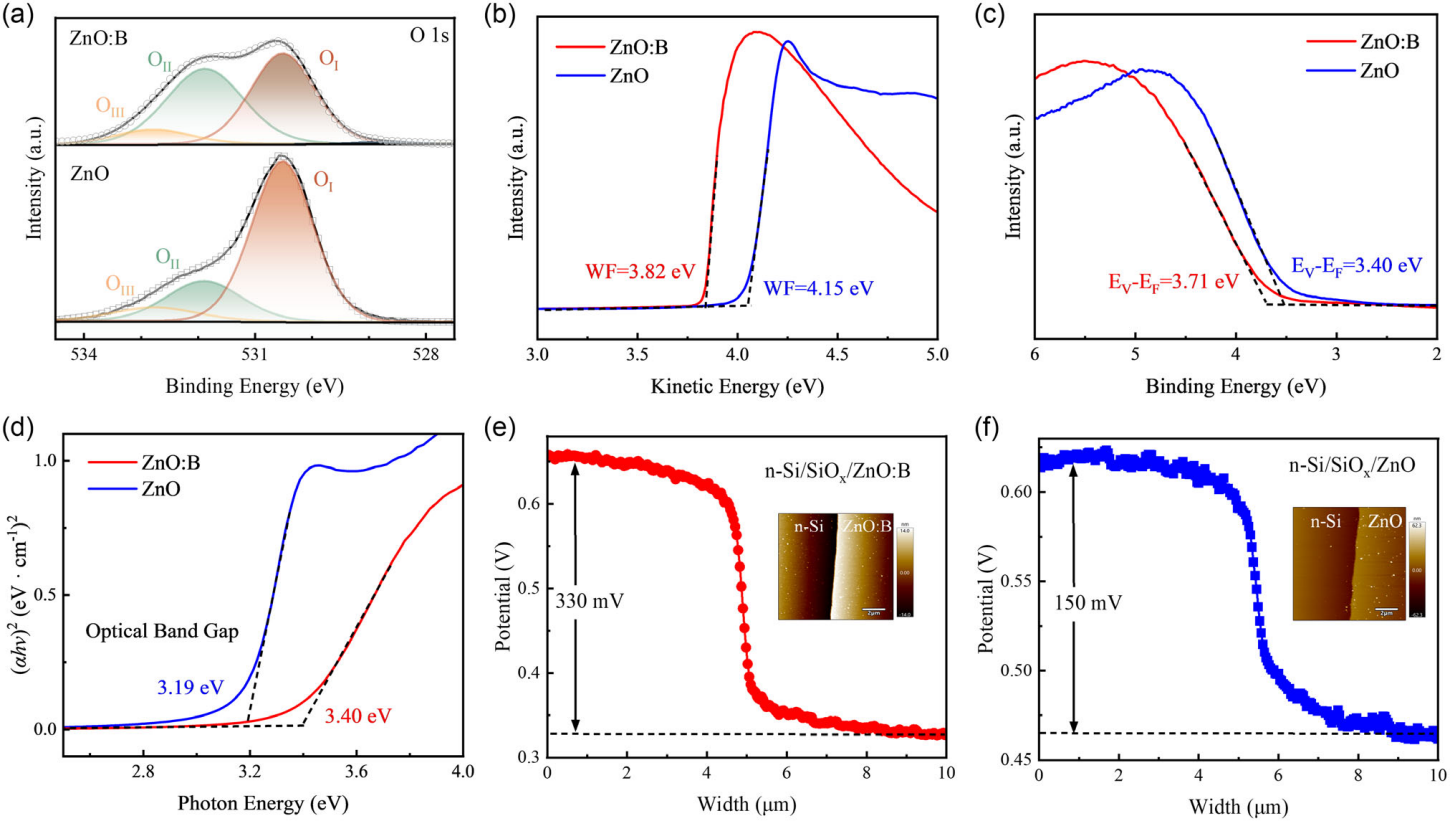
Composition and electronic structure of ZnO:B film (with Zn:B cycle ratio of 10:1). a) XPS core‐level spectra of O1s of ZnO:B and ZnO films. b) The secondary‐electron cutoff spectrum and c) valence band spectrum of ZnO:B and ZnO films by UPS measurements. d) Optical absorption spectrum of ZnO:B and ZnO films. The surface potential of the e) n‐Si/ZnO:B and f) n‐Si/ZnO contact measured by SKPM, the insets show the surface morphology measured by atomic force microscope (AFM).

To further investigate the influence of B doping on the elemental composition and electronic structure of ZnO:B film, XPS and ultraviolet photoelectron spectroscopy (UPS) measurement were conducted on ZnO:B film with a Zn:B cycle ratio of 10:1, compared to pristine ZnO film, as shown in **Figure** **2**. The elemental analysis primarily focuses on the core‐level spectra of O 1*s* (Figure 2a). By Gaussian deconvolution, the O 1*s* peaks were split into three parts, O_I_, O_II_, and O_III_ centered at 530.5, 531.9, and 532.8 eV, respectively, for both ZnO and ZnO:B films. Among these peaks, the lowest one (O_I_) represents lattice O, while the medium one (O_II_) is related to oxygen vacancy in the film.^[^ ^37^ ^]^ Compared to the pristine ZnO film, the O 1*s* core‐level spectra of ZnO:B film show a higher O_II_ peak and lower O_I_ peak, indicating that B doping can promote the generation of oxygen vacancy, effectively improving the electrical properties of the film. The highest binding energy peak (O_III_), related to the loosely bound oxygen adsorbed on the surface, shows no significant difference between the pristine ZnO and ZnO:B film.^[^ ^38^ ^]^

**Figure 3:**
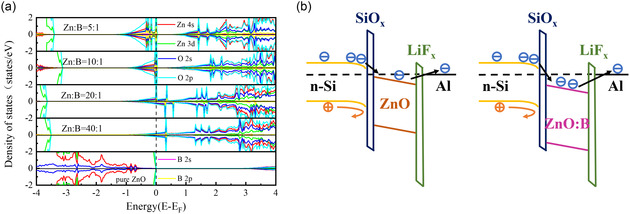
a) DFT‐calculated DOS plots of pristine ZnO film and B‐doped ZnO film (Zn:B atomic ratios of 40:1, 20:1, 10:1, and 5:1). b) Band diagram of n‐Si/SiO_*x*_/ZnO/LiF_*x*_/Al contact and n‐Si/SiO_*x*_/ZnO:B/LiF_*x*_/Al contact.

UPS secondary electron cutoff analysis was used to determine the electronic structure of the thin films. After doping with B element, the work function of the ZnO film decreased from 4.15 to 3.82 eV, as shown in Figure 2b. This lower work function of ZnO:B film is crucial for achieving lower contact resistivity on the c‐Si surface by facilitating a downward bending at the c‐Si surface. Figure 2c displays the valence band of the ZnO and ZnO:B films, which are measured to be 3.40 and 3.71 eV from the Fermi energy, respectively. The optical bandgaps of the deposited ZnO and ZnO:B films were determined to be 3.19 and 3.40 eV, respectively, based on the Tauc plot of the films shown in **Figure** **3**d. These results indicate that the Fermi level of the ZnO film is shifted into the conduction band, suggesting the delocalization of the electrons and demonstrating that effective B doping enhances the carrier transport performance of the ZnO film.

To further investigate the influence of B element doping on the position shift of the energy band in ZnO film, detailed density functional theory (DFT) calculations were performed using varying concentrations of B atoms to replace Zn atoms in the ZnO lattice, as illustrated in Figure 3a. Figure S4, Supporting Information, presents the ZnO supercells used in these simulations, with B atoms at different doping ratios. The calculated band structure plots for both the pristine ZnO film and concentrated‐doped ZnO films are shown in Figure S5, Supporting Information. For the pristine ZnO film, the density of states (DOS) indicates a defect‐free bandgap of 2.85 eV, which is slightly lower than the experimentally measured value of 3.24 eV. This discrepancy arises from the bandgap underestimation typical when using Perdew–Burke–Ernzerhof functionals in DFT calculations. The valence band primarily comprises O 2*p* states with minor contributions from Zn 3*d* states, whereas the conduction band mainly consists of Zn 3*d* states with a small contribution from O 2*p* states. Incorporating B dopants alters the electronic properties, such as introducing defect states, thereby modifying bandgap energies. The ZnO:B films exhibit a reduced fundamental bandgap (the difference of conduction band minimum (CBM) and valence band maximum (VBM)). Both CBM and VBM shift to lower energy levels, indicating significant resonance and bonding tendencies among the Zn 4*s*, O 2*p*, B 2*s*, and O 2*p* orbitals in the CBM. Compared to the pristine ZnO film, the ZnO:B films show a significant upward shift of the Fermi level, which moves into the conduction band. This indicates that B doping effectively enhances the electrical properties of ZnO films.^[^ ^39^ ^]^

**Figure 4:**
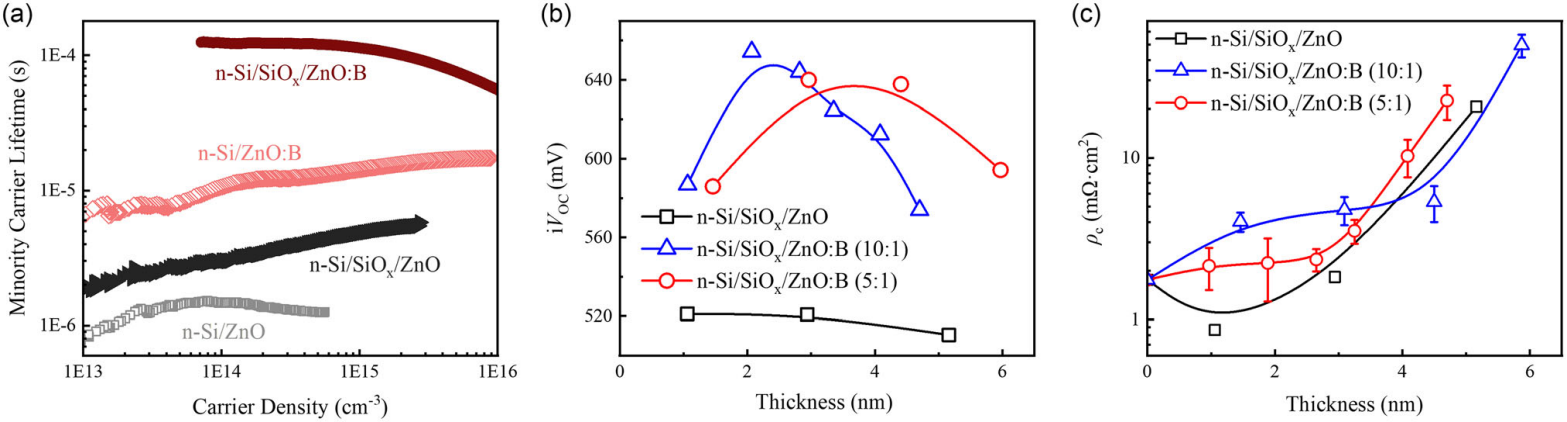
Passivation and contact properties of c‐Si/ZnO:B contact. a) Effective minority carrier lifetime of symmetrically structured c‐Si/ZnO, c‐Si/SiO_*x*_/ZnO, c‐Si/ZnO:B, and c‐Si/SiO_*x*_/ZnO:B contacts. ZnO and ZnO:B thickness‐dependent b) *iV*_OC_ and c) contact resistivity of c‐Si/SiO_*x*_/ZnO, c‐Si/SiO_*x*_/ZnO:B (Zn:B atomic ratio of 10:1 and 5:1) contacts.

To visualize the interfacial potential changes of ZnO and ZnO:B films on the c‐Si surface, scanning Kelvin probe microscopy (SKPM) was employed, as shown in Figure 2e,f. For the sample with the ZnO film deposited, the surface potential difference of ZnO‐coated area and bare c‐Si surface is about 150 mV (Figure 2f). In contrast, for the ZnO:B deposited sample, the surface potential difference was ≈330 mV (Figure 2e), indicating a lower work function for the ZnO:B film compared to the pristine ZnO film. Based on the energy band model measured above, **Figure** **4**b illustrates the energy band diagrams of n‐Si/SiO_*x*_/ZnO/LiF_*x*_/Al and n‐Si/SiO_*x*_/ZnO:B/LiF_*x*_/Al contacts. The lower work function of ZnO:B induces a better downward bending of the n‐Si energy band, resulting in an enrichment of electrons at the c‐Si surface.^[^ ^40^ ^]^ Moreover, a larger valence band offset of the ZnO:B film from the c‐Si can effectively shield holes, thereby mitigating surface recombination and exhibiting superior electron extraction capabilities.

### 2.2 Passivation and Contact Properties of ZnO:B/n‐Si Contact

The effective minority carrier lifetime *τ*_eff_ and contact resistivity *ρ*_c_ measurements were utilized to evaluate the electron‐selective passivating contact performance of ZnO:B film on c‐Si, as shown in Figure 4. The *τ*_eff_ was measured using symmetrically deposited samples and the *ρ*_c_ was measured using the transfer‐length method (TLM) model.^[^ ^41^ ^]^ Figure 4a shows the *τ*_eff_ of symmetrically structured c‐Si/ZnO and c‐Si/ZnO:B contacts with and without UV/O_3_‐oxidized SiO_*x*_ interlayer. Unlike c‐Si/ZnO contact, which exhibits negligible passivation properties, the c‐Si/ZnO:B contact demonstrates better passivation properties. Upon inserting an ultrathin UV/O_3_ oxidized SiO_*x*_ interlayer (≈1.5 nm), the *τ*_eff_ increased to over 120 μs. Noting that this improved passivation performance is achieved through a postannealing process, Figure S6, Supporting Information, illustrates the effect of the postannealing process on the passivation properties of c‐Si/SiO_*x*_/ZnO:B contacts. As evidenced by the XPS core‐level spectra of O 1*s* for the as‐deposited and postannealed ZnO:B films (Figure S7, Supporting Information), this improved passivation property is attributed to the increased O vacancies in the ZnO:B film during annealing process. The ZnO and ZnO:B thickness‐dependent implied open‐circuit voltages (*iV*_OC_) for both c‐Si/SiO_*x*_/ZnO and c‐Si/SiO_*x*_/ZnO:B contacts are investigated in Figure 4b. A thin ZnO:B film with thickness about 3 nm supports the best passivation properties. Further increasing the thickness of ZnO:B film leads to a decline in passivation performance, which may be attributed to the influence of internal stress during film deposition.

To further explore the contact properties of ZnO:B film on c‐Si, Figure 4c shows the ZnO:B thickness‐dependent contact resistivities of c‐Si/SiO_*x*_/ZnO:B contact. For reference, c‐Si/SiO_*x*_/ZnO contact is also included. Low‐work function LiF_*x*_/Al metal electrodes were applied to all samples used for contact resistivity measurements to ensure Ohmic contact, as shown in Figure S8, Supporting Information. Even when the thickness of ZnO:B films increased to 5 nm, a stable contact resistivity of less than 10 mΩ cm^2^ was obtained. The influence of B doping concentration on the passivation contact properties of ZnO:B film is also present in Figure 4b,c, showing a consistent trend.

### 2.3 Photovoltaic Performance of c‐Si Solar Cells with ZnO:B Passivating Contact

**Figure 5:**
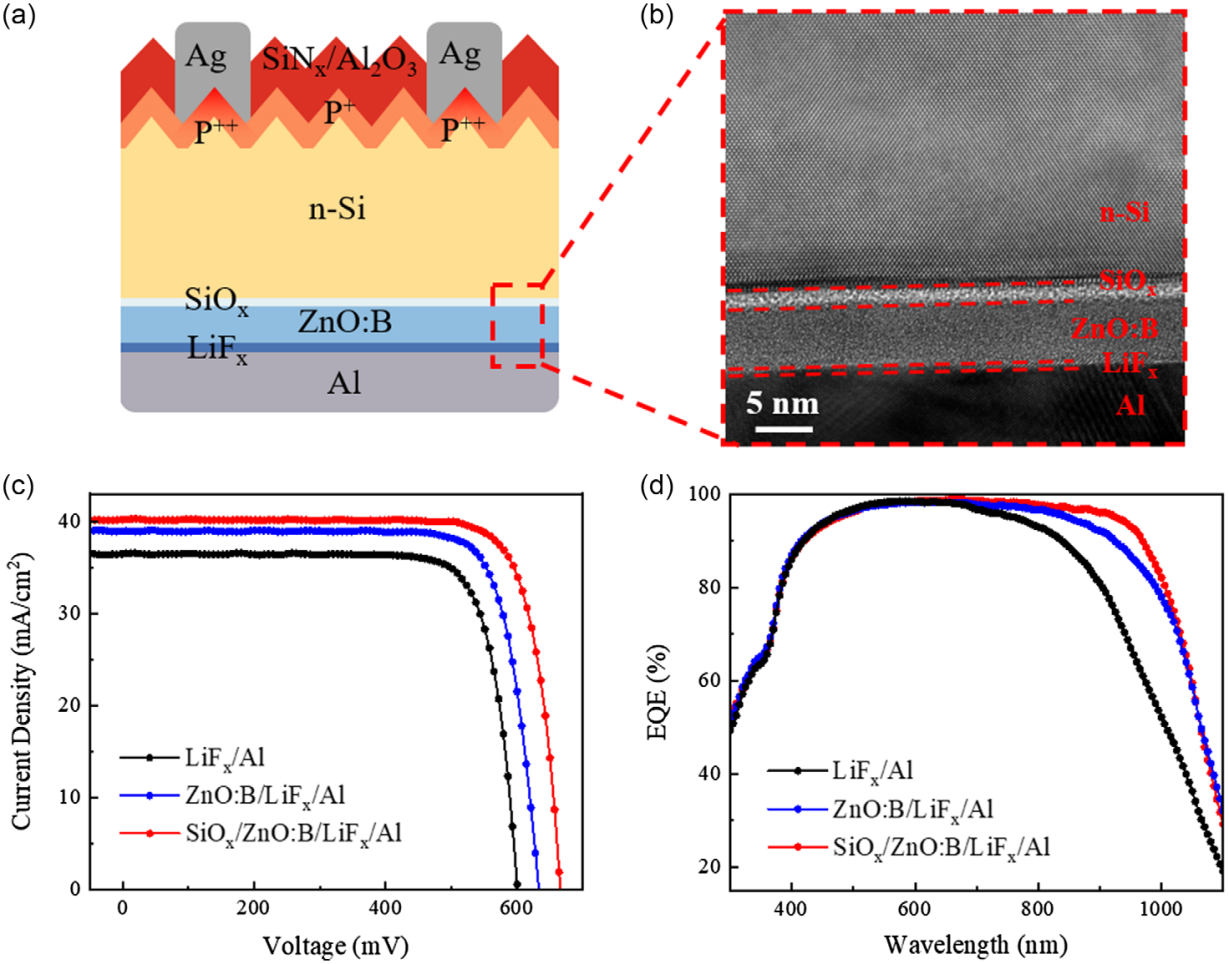
Proof‐of‐concept c‐Si solar cells with ZnO:B rear‐side passivating contact. a) The schematic of n‐type c‐Si solar cell with full‐area SiO_*x*_/ZnO:B/LiF_*x*_/Al ESCs. b) HRTEM image of c‐Si/SiO_*x*_/ZnO:B/LiF_*x*_/Al contact. c) Light *J–V* curves and d) EQE curves of n‐type c‐Si solar cells with LiF_*x*_/Al, ZnO:B/LiF_*x*_/Al, and SiO_*x*_/ZnO:B/LiF_*x*_/Al rear contacts under standard test conditions.

To verify the potential of ZnO:B film as an ESC layer in solar cells, full‐area electron‐selective ZnO:B contacts were integrated into proof‐of‐concept n‐type c‐Si solar cells on the rear‐side. The schematic structure is illustrated in **Figure** **5**a. High‐resolution transmission electron microscopy (HRTEM) and energy‐dispersive X‐ray spectroscopy imaging was employed to characterize the structure of c‐Si/SiO_*x*_/ZnO:B/LiFx/Al contact interface, as shown in Figure 5b and S9, Supporting Information, where a clear hierarchical structure can be observed. Three different rear‐side ESC structures were used for comparison, including ZnO:B/LiF_*x*_/Al, SiO_*x*_/ZnO:B/LiF_*x*_/Al, and direct LiF_*x*_/Al contact as a reference. A commercially available boron‐diffused p^+^ emitter coated with Al_2_O_3_/SiN_*x*_ passivating/antireflection stack and Ag screen‐printed electrode was applied as the front‐side contact in these solar cells.

The characteristic current density–voltage (*J–V*) curves of n‐type c‐Si solar cells with LiF_*x*_/Al, ZnO:B/LiF_*x*_/Al, and SiO_*x*_/ZnO:B/LiF_*x*_/Al electron‐selective rear contacts under standard test conditions are displayed in Figure 5c. The corresponding PV parameters are summarized in **Table** **1**. The direct deposition of LiF_*x*_/Al as the rear‐side contact resulted in a relatively low PCE of 17.4%, with an open circuit‐voltage (*V*_OC_) of 598.8 mV, a *J*_SC_ of 36.5 mA cm^−2^, and a fill factor (FF) of 79.5%. This poor PV output can be ascribed to the high carrier recombination loss at the rear c‐Si/LiF_*x*_/Al interface. After inserting a thin ZnO:B interlayer between c‐Si and LiF_*x*_/Al electrode, the PV performance can be improved with a PCE of 19.9%, a *V*_OC_ of 631.8 mV, a *J*_SC_ of 39.1 mA cm^−2^, and FF of 81.1%. Introducing a thin UV/O_3_ oxidized SiO_*x*_ interlayer between c‐Si and ZnO:B interface further enhanced the PCE to over 22.0%, with a maximum *V*_OC_ of 665.2 mV, a *J*_SC_ of 40.2 mA cm^−2^, and FF of 82.4%. This substantial improvement in PV performance can be attributed to the enhanced surface passivation and reduced contact resistance between c‐Si and LiF_*x*_/Al electrode, as discussed in Figure 4. Table S1, Supporting Information, also compares the PV performances of c‐Si solar cells using metal compounds as electron‐selective transport layers and SiO_*x*_ as a passivating interlayer, showing that the use of SiO_*x*_/ZnO:B ESCs results in superior PV output behavior.

**Table 1:** PV parameters of champion n‐type c‐Si solar cells with LiF_*x*_/Al, ZnO:B/LiF_*x*_/Al, and SiO_*x*_/ZnO:B/LiF_*x*_/Al rear contacts under standard test conditions.

Rear contacts	*V*_OC_ [mV]	*J*_SC_ [mA cm^−2^]	FF [%]	PCE [%]
LiF_*x*_/Al	598.8	36.5	79.5	17.4
ZnO:B/LiF_*x*_/Al	631.8	39.1	81.1	19.9
SiO_*x*_/ZnO:B/LiF_*x*_/Al	665.2	40.2	82.4	22.0

The *J*_SC_ values of c‐Si solar cells using LiF_*x*_/Al, ZnO:B/LiF_*x*_/Al, and SiO_*x*_/ZnO:B/LiF_*x*_/Al electron‐selective rear contacts are further validated by the external quantum efficiency (EQE) measurement, as shown in Figure 5d. A significant enhancement of the EQE values, especially in the long‐wavelength range, is observed for the SiO_*x*_/ZnO:B/LiF_*x*_/Al contact device. This improvement indicates reduced carrier recombination losses and enhanced electron‐selective collection ability at the rear side.

**Figure 6:**
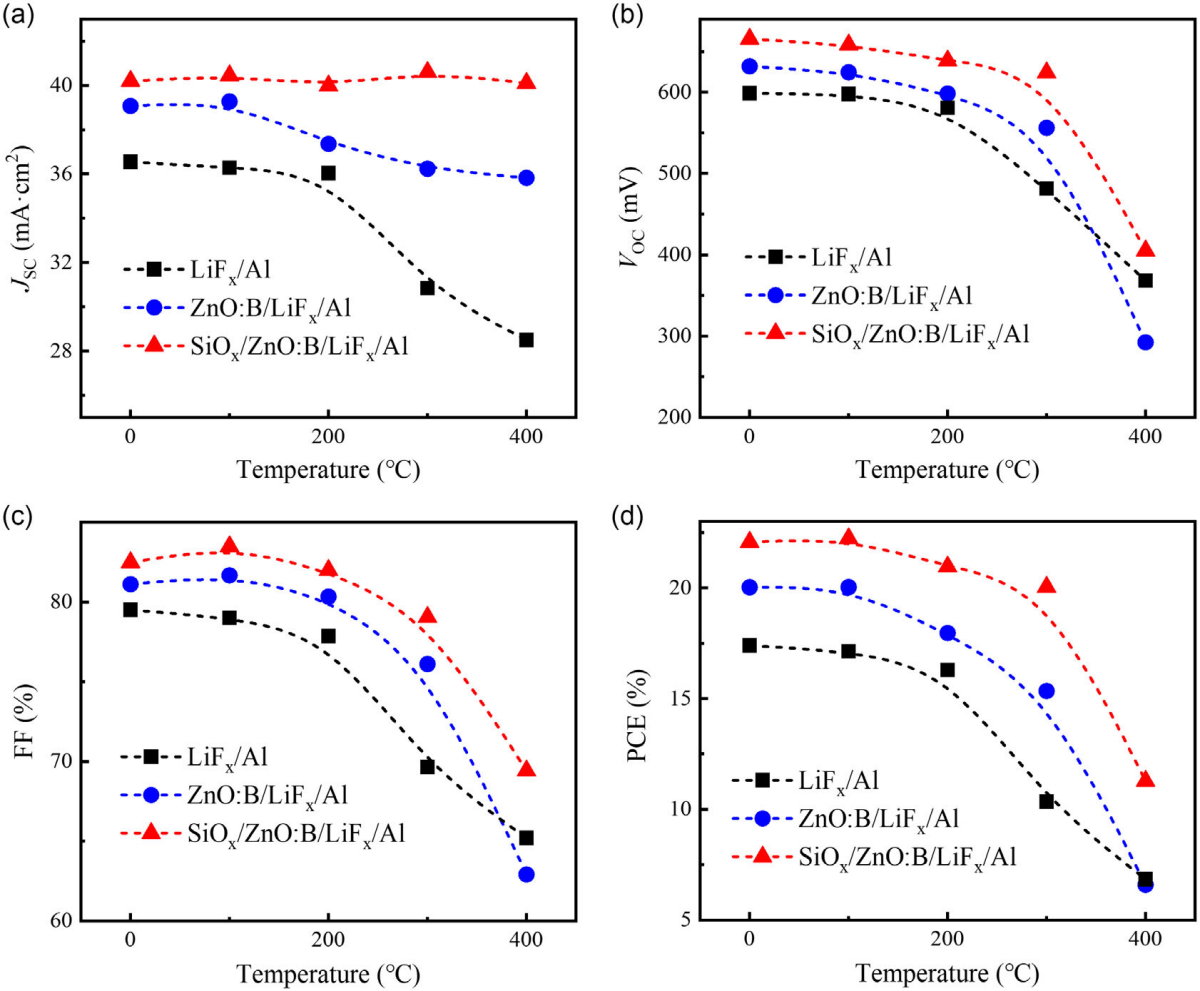
Thermal stability of c‐Si solar cells. a) *J*_SC_, b) *V*_OC_, c) FF, and d) PCE of the c‐Si solar cells using LiF_*x*_/Al, ZnO:B/LiF_*x*_/Al, and SiO_*x*_/ZnO:B/LiF_*x*_/Al contacts under different annealing temperatures in air.

To investigate the thermal stability, the abovementioned c‐Si solar cells with LiF_*x*_/Al, ZnO:B/LiF_*x*_/Al, and SiO_*x*_/ZnO:B/LiF_*x*_/Al electron‐selective rear contacts were annealed in air for 30 min at different temperatures. The corresponding PV output parameters are presented in **Figure** **6**. Compared with directly LiF_*x*_/Al or ZnO:B/LiF_*x*_/Al contact, solar cells featuring SiO_*x*_/ZnO:B/LiF_*x*_/Al rear side contact demonstrate better thermal stability, especially in terms of *J*_SC_ parameter. The decrease in PCE primarily stems from the decline in *V*_OC_, which may be attributed to the diffusion of Al element from the metal electrode to the c‐Si interface, thereby degrading the passivating contact properties.^[^ ^42^ ^]^ Additionally, only a slight decrease in PCE was observed for the solar cells using ZnO:B as the electron‐selective passivating contact at annealing temperature below 300 °C. However, when the annealing temperature exceeds 300 °C, the PV performances declines significantly, likely due to instability of LiF_*x*_ layer at high temperatures and substantial Al diffusion into the c‐Si interface.^[^ ^43^ ^]^

## 3 Conclusion

In summary, we have demonstrated that SiO_*x*_/ZnO:B contact can serve as effective electron‐selective passivating contact structures for c‐Si solar cells. The successful doping of B in ZnO film via the ALD process significantly enhances their electrical properties and energy band structure. This leads to a considerable reduction in contact resistivity and improved surface passivation when in contact with c‐Si. A satisfied PCE of over 22.0% was achieved in proof‐of‐concept n‐type c‐Si solar cells utilizing full‐area SiO_*x*_/ZnO:B/LiF_*x*_/Al electron‐selective passivating contacts on the rear side. These results highlight an effective and straightforward approach to design high‐efficiency and stable c‐Si solar cells with metal oxide carrier‐selective passivating contact architectures.

## 4 Experimental Section

4.1

4.1.1

##### Deposition and Characterization of ZnO:B Film

ZnO:B films were deposited by ALD process, using diethylzinc (DEZ, purchased from Sigma‐Aldrich with purity over 99.999%) and triisopropyl borate (TIB, purchased from Sigma‐Aldrich with purity over 99.999%) precursors as Zn and B source and DI H_2_O as O source at a deposition temperature of 150 °C. B doping was achieved by inserting a B cycle into the Zn/O cycle in each Zn:B supercycle and the B‐doping concentration was controlled using different cycle ratios (Zn:B) of 40:1, 20:1, 10:1, and 5:1. The thickness of as‐deposited ZnO:B films was calibrated by ellipsometry. The chemical composition and the work function of the films were characterized by XPS/UPS measurements with a Thermo Scientific Escalab 250Xi spectrometer using the Al Kα X‐ray source (*hν* = 1486.6 eV). The electrical properties and crystal structure of the ZnO:B films were obtained by Hall test and XRD measurement, respectively, with thickness fixed at about 60 nm. DFT calculations were used to examine the changes in energy band structure and the theoretical electronic properties of the ZnO:B films.

##### Passivation and Contact Measurement

Double‐side pyramid‐textured n‐type c‐Si wafers (100) with a thickness of 135 μm and resistivity of 0.4–0.8 Ω cm were used for passivation and contact characterization. After standard Radio Corporation of America (RCA) cleaning and following dilute hydrofluoric acid (HF, c2% concentration) dip, ZnO:B films with different thicknesses were deposited on both sides of c‐Si. A thin UV/O_3_ SiO_*x*_ layer was introduced before ZnO:B deposition and the temperature UV–O_3_ treatment was carried out at 50 °C with a mercury lamp (185 and 245 nm with 25 mW cm^−2^) for 300 s. For the contact measurement samples, a 1.5 nm/300 nm LiF_*x*_/Al electrode was deposited using thermal evaporation through a TLM‐patterned shadow mask, at the rate of 0.1 and 1 Å s^−1^, respectively. The effective minority carrier lifetimes of samples were characterized by photoconductance decay (Sinton WCT 120).

##### Solar Cell Device

Solar cells were demonstrated using a phosphorus‐doped n‐type c‐Si wafer, with a full‐area p^+^ and local heavily doped p^++^ emitter on the front side. Al_2_O_3_ (20 nm)/SiN_*x*_ (55 nm) stacks were used as passivating and antireflection layer. Front metallization was realized by screen printing of silver paste and the subsequent firing process. For the rear contact, a thin SiO_*x*_ passivating interlayer was prepared by UV/O_3_ method mentioned above. Full‐area ZnO:B (Zn:B = 10:1) films with thickness of 3 nm were deposited by ALD, followed with FGA and the deposition of 1.5 nm/300 nm LiF_*x*_/Al electrode. The PV performances were characterized by a solar simulator (Sinton Instruments) under standard test conditions (AM 1.5G, 100 mW cm^−2^) with a fixed exposure area of 2.56 cm^2^. The test temperature was controlled at 25 ± 0.5 °C. HRTEM of c‐Si/SiO_*x*_/ZnO:B/LiF_*x*_/Al contact was obtained from FEI Titan Tecnai F20 TEM microscope operated at 200 kV.

## Conflict of Interest

The authors declare no conflict of interest.

## Author Contributions

**Zheng Li**: Data curation (equal); Investigation (equal); Writing—original draft (lead). **Anzhi Xie**: Data curation (equal); Writing—original draft (equal). **Qingxian Nong**: Software (equal); Writing—original draft (supporting). **Yiwei Sun**: Writing—original draft (supporting). **Haihuai Cai**: Writing—original draft (supporting). **Zhexi Chen**: Writing—original draft (supporting). **Jian He**: Conceptualization (lead); Project administration (lead); Resources (lead); Supervision (lead); Writing—review & editing (lead). **Pingqi Gao**: Supervision (equal).

## Supporting information

Supplementary Material

## Data Availability

The data that support the findings of this study are available from the corresponding author upon reasonable request.
