# Supplementary material for: Boron‐Doped Zinc Oxide Electron‐Selective Contacts for Crystalline Silicon Solar Cells with Efficiency over 22.0%
Source: Small Sci. 2024 Sep 22;4(11):2400168. doi: 10.1002/smsc.202400168 (PMC11935270; doi:10.1002/smsc.202400168)
Supplement: Supplementary file 1 — Supplementary Material [file SMSC-4-2400168-s001.pdf]

Supporting Information

Boron-Doped Zinc Oxide Electron-Selective Contacts for Crystalline Silicon Solar Cells with Efficiency over 22.0%

Zheng Li[1#], Anzhi Xie[1#], Qingxian Nong[1], Yiwei Sun[1], Haihuai Cai[1], Zhexi Chen[1], Jian He[1, 2, *], Pingqi Gao[1, 2, *]

[1] School of Materials, Shenzhen Campus of Sun Yat-sen University, No. 66, Gongchang Road, Shenzhen, Guangdong 518107, P.R. China

[2] Institute for Solar Energy Systems, Guangdong Engineering Technology Research Center for Sustainable Photovoltaic Technology and Equipment, State Key Laboratory of Optoelectronic Materials and Technologies, Sun Yat-sen University, Guangzhou, 510275, P.R. China

[#] These authors contribute equal to this paper

[*] E-mail: hejian7@mail.sysu.edu.cn; gaopq3@mail.sysu.edu.cn

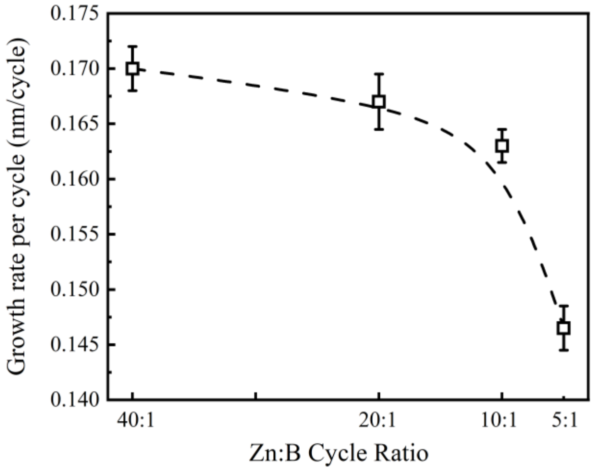

Figure S1. The deposition rate of ZnO:B films at different Zn:B cycle ratio.

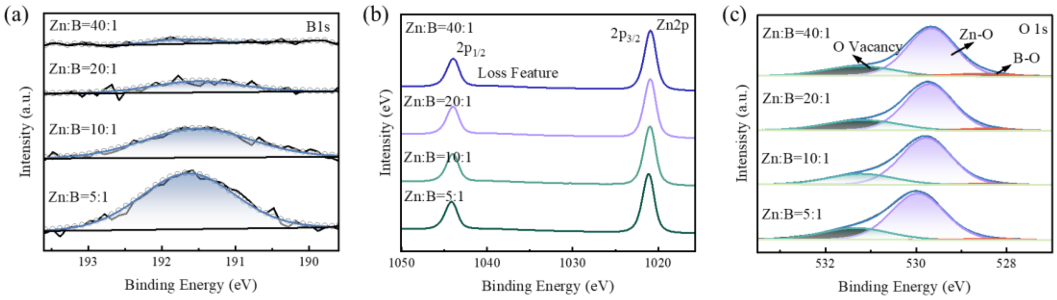

Figure S2. XPS Core-level spectra of (a) B1s, (b) Zn 2p, (c) O 1s of ZnO:B film with different Zn:B cycle ratio.

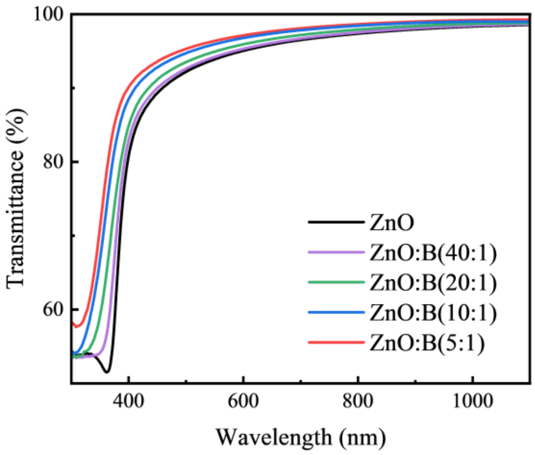

Figure S3. Transmittance curves of ZnO:B at different ratio.

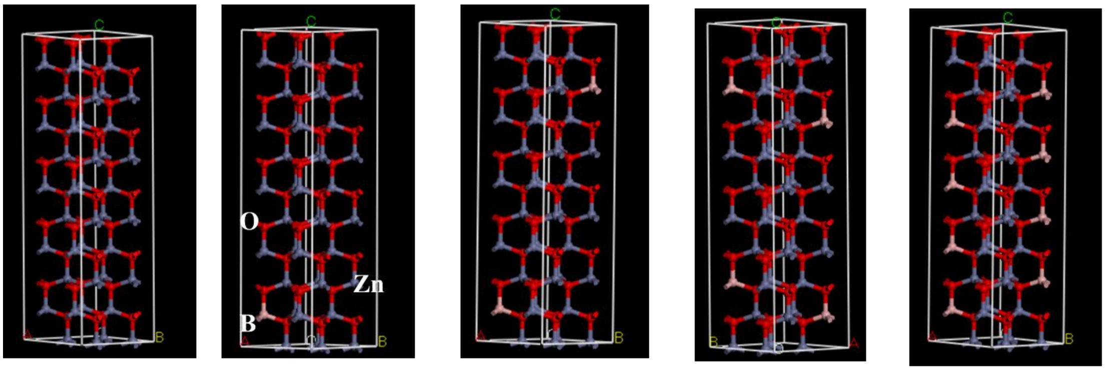

Pristine ZnO　Zn:B = 40:1　Zn:B = 20:1　Zn:B = 10:1　Zn:B = 5:1

Figure S4. ZnO supercell with B as foreign atom at different ratio.

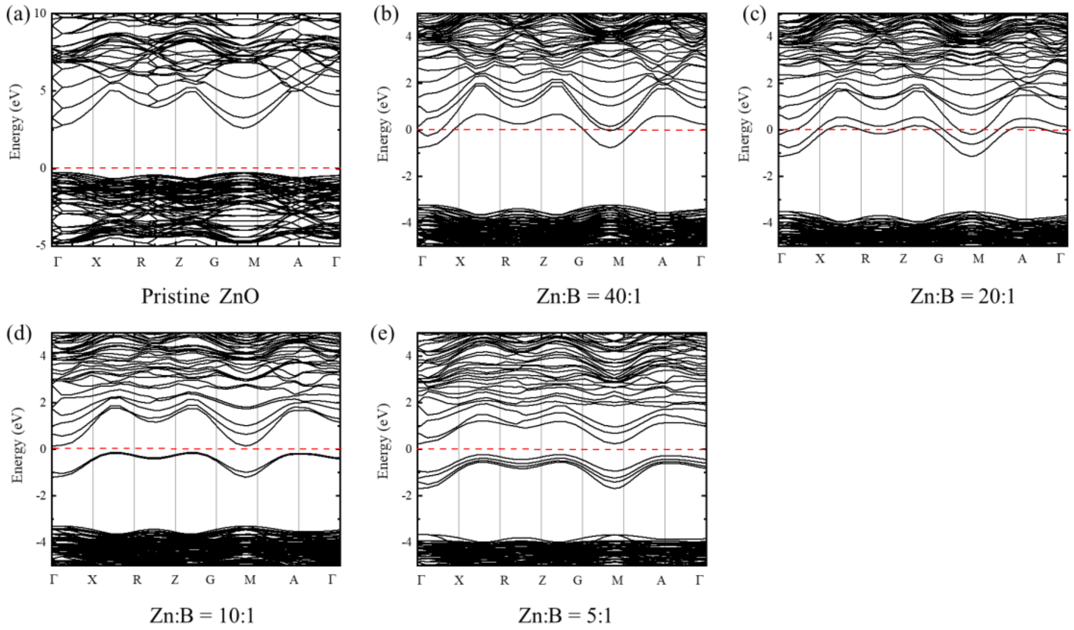

Figure S5. Band structure plots along the Γ-X-R-Z-G-M-A-Γ path for (a) prostine ZnO, ZnO:B with Zn:B atom ratio of (b) 40:1, (c) 20:1, (d) 10:1, and (e) 5:1. The red dash line in each figure is the Fermi level.

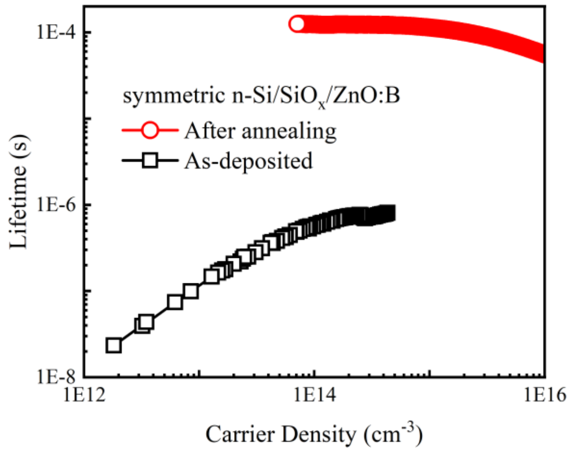

Figure S6. Effective minority carrier lifetime of symmetrically structured c-Si/SiO_x/ZnO:B contact before and after annealing

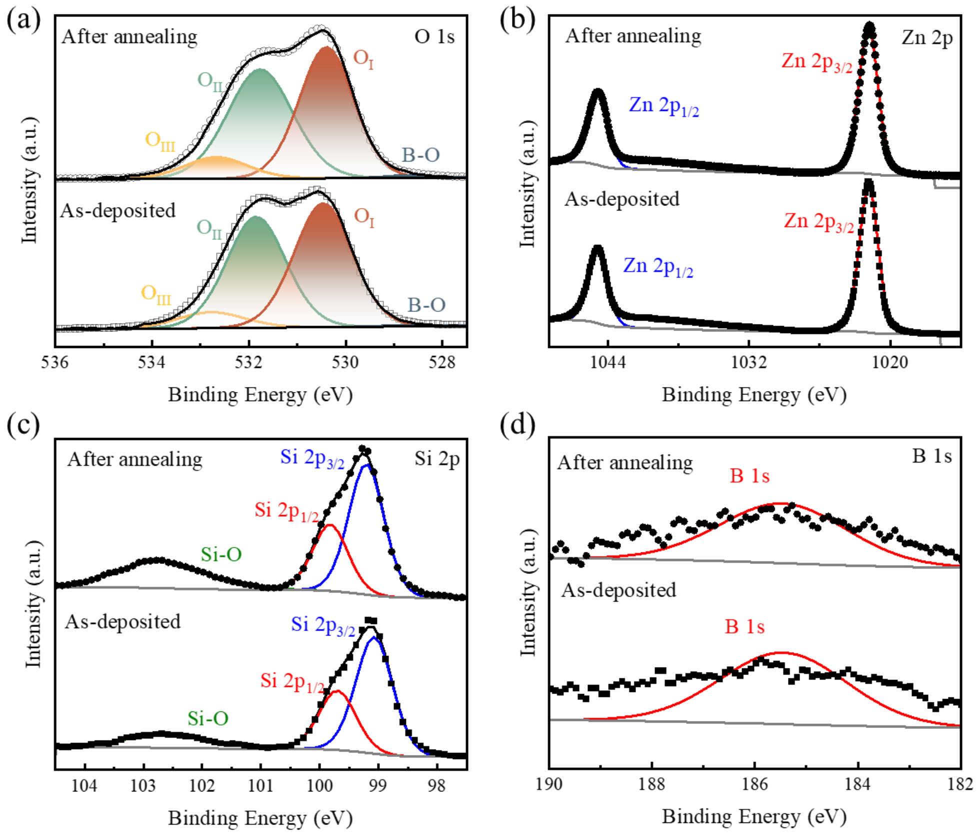

Figure S7. XPS Core-level spectra of (a) O1s, (b) Zn 2p, (c) Si 2p, and (d) B 1s of ZnO:B film before and after annealing.

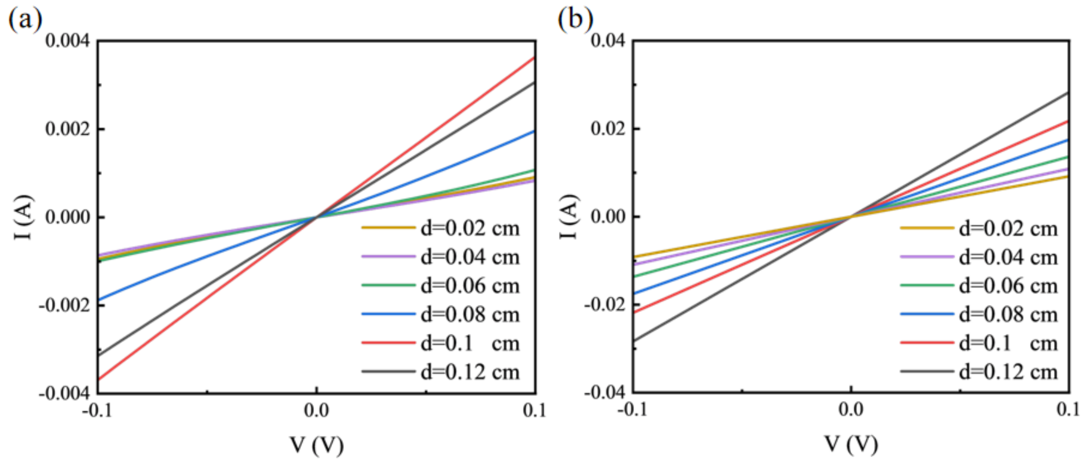

Figure S8. Contact resistance result of (a)without LiF_x and (b)with LiF_x.

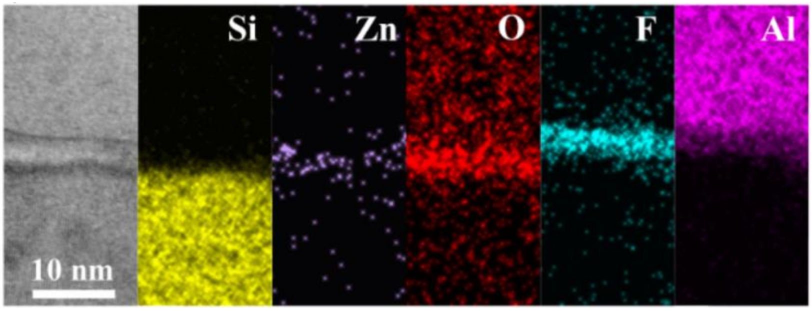

Figure S9. High-resolution transmission electron microscopy (HR-TEM) and Energy Dispersive X-Ray Spectroscopy (EDX) imaging of c-Si/SiO_x/ZnO:B/LiF_x/Al.

Table S1. Recently reported c-Si solar cells using SiO_x/metal compounds stack as electron selective passivating contacts.

**Material**	**ESCs**	J_{SC} **[mA/cm^2]**	V_{OC} **[mV]**	**FF [%]**	**PCE [%]**	**Ref.**
TiO_x	n-Si/SiO_x/TiO_x/Al/Ag	39.8	674	82.5	22.1	[1]
TiN_x	n-Si/SiO_x/TiN_x/Al	37.9	644	81.9	20.0	[2]
TbF_x	n-Si/SiO_x/TbF_x/Al	38.5	675	84.1	21.9	[3]
SnO_x	n-Si/SiO_x/SnO_x/Mg	35.23	605	66.18	14.11	[4]
$AlCl_x$	n-Si/SiO_x/$AlCl_x$/Al	39.3	620.1	81.2	19.8	[5]
AlF_x	n-Si/SiO_x/AlF_x/Al	39.6	645.3	82.3	21.0	[5]
SrO_x	n-Si/SrO_x/Mg/Al	39.1	619	82.8	20.0	[6]
TiOx:Ta	n-Si/SiO_x/TiOx:Ta /LiF_x/Al	39.76	653.5	83.07	21.58	[7]
BaOx	n-Si/SiO_x/BaO_x /LiF_x/Ca/Al	39.2	625.7	83.5	20.5	[8]
ZnO:B	n-Si/SiO_x/ZnO:B /LiF_x/Al	40.2	665.2	82.4	22.0	This Work

Reference

[1] Yang X, Weber K, Hameiri Z, et al. Industrially feasible, dopant-free, carrier-selective contacts for high-efficiency silicon solar cells. Progress in photovoltaics: Research and Applications, 2017, 25(11): 896-904.
[2] Yang X, Liu W, De Bastiani M, et al. Dual-function electron-conductive, hole-blocking titanium nitride contacts for efficient silicon solar cells. Joule, 2019, 3(5): 1314-1327.
[3] Wei H, Meng L, Liu Z, et al. A Novel Ultra-Low Work Function TbFx for High Efficiency Dopant-Free Silicon Solar Cells. Small, 2023, 19(37): 2300879.
[4] Liu M, Zhou Y, Dong G, et al. SnO2/Mg combination electron selective transport layer for Si heterojunction solar cells. Solar Energy Materials and Solar Cells, 2019, 200: 109996.
[5] Gao K, Xing C, Xu D, et al. Aluminum Halide-Based Electron-Selective Passivating Contacts for Crystalline Silicon Solar Cells. Small, 2024: 2310352.
[6] Xing C, Gu W, Gao K, et al. Electron-Selective Strontium Oxide Contact for Crystalline Silicon Solar Cells with High Fill Factor. Solar RRL, 2023, 7(9): 2201100.
[7] Zhang L, Qiu J, Cheng H, et al. Low-temperature Ta-doped TiOx electron-selective contacts for high-performance silicon solar cells. Solar Energy Materials and Solar Cells, 2024, 266: 112703.
[8] Xing C, Gu W, Xiang Z, et al. Post-annealing-free BaOxFy/LiF-based stack electron-selective contacts for high efficiency crystalline silicon solar cells featuring ultra-low contact resistivity. Chemical Engineering Journal, 2024, 481: 148568.
